# Acute pulmonary melioidosis presenting with multiple bilateral cavitary lesions in a healthy young adult: an authentic case report from Sri Lanka

**DOI:** 10.1186/s13104-016-2168-2

**Published:** 2016-07-22

**Authors:** Chathuranga Lakmal Fonseka, Sampath Rukshani Galappaththi, Anuradha Illagatilaka, Dulani Dasanayake, Nirmali Tissera

**Affiliations:** General Medicine, National Hospital, Colombo, Sri Lanka; National Hospital, Colombo, Sri Lanka

**Keywords:** Melioidosis, Cavitary lesions, Broncho-alveolar lavage

## Abstract

**Background:**

Melioidosis is an emerging infectious disease in Sri Lanka. This disease usually develops in a host with an immunocompromised state. Acute pulmonary melioidosis commonly presents as a lobar consolidation with or without pulmonary nodules or abscesses involving the upper lobes of the lungs.

**Case presentation:**

We report a young healthy female who does not have known risk factors or immunocompromised state, presented with bilateral multiple cavitary lesions involving all three zones of the lungs. She used to involve in home gardening. Her initial relevant microbiological investigations were all negative. The diagnosis of melioidosis was made by broncho-alveolar lavage fluid culture positivity combined with a highly positive antibody titre. She showed dramatic response to intravenous high dose Meropenem.

**Conclusions:**

Melioidosis should be suspected early in patients with acute pulmonary involvement who show poor response to conventional antibiotics, even in the absence of known risk factors for disease. Other than known occupational exposures, household exposures such as home gardening should also be considered as a possible mode of exposure.

## Background

Melioidosis is an uncommon yet potentially fatal emerging infectious disease in Sri Lanka. Although Sri Lanka is not considered as a country where melioidosis is endemic, an increasing number of cases at an alarming rate have been reported recently [1]. It is transmitted either by inhaling dust contaminated with bacteria or contaminated soil coming into contact with abraded areas of skin [1, 2]. It is exceedingly uncommon for the young healthy hosts to develop the acute fulminant form of the disease; but those with diabetes, renal impairment, heart disease, malnutrition, chronic pulmonary disease, excess alcohol consumption and patients with HIV, commonly develops the acute fulminant form, often complicated with septicaemia and septic shock [3–5]. In certain instances, melioidosis could remain quiescent for a long time and can recur when the host immune response gets impaired [6].

Melioidosis clinically can present as a disseminated form or localized disease. The disseminated form is characterized by an acute, rapidly progressive course with septicaemia or septic shock which could progress into respiratory failure and adult respiratory distress syndrome. These individuals may frequently have concurrent superficial cutaneous abscesses and/or multiple liver and splenic abscesses. Occasionally, these patients may present with fever of unknown origin [7]. Localized form of melioidosis can involve many sites both pulmonary and also extra pulmonary involving joints, brain etc. [8].

Acute pulmonary melioidosis usually present with lobar or segmental consolidation or multi-lobar pulmonary infiltrates which could rapidly progress into cavity formation. The upper lobes are most frequently affected up to 95 %, resembling the radiological appearance of tuberculosis. However, the lung cavities in melioidosis do not often show an air–fluid level, unless they are very large. Another frequent pattern is the presence of solitary or multiple pulmonary nodules. Radiological presence of pneumatoceles or pleural effusions favors the diagnosis of *Staphylococcal* pneumonia. The rapid spread of infection to other organs forming visceral abscesses also favors melioidosis over disseminated tuberculosis [9, 10].

Definitive diagnosis of melioidosis is made by culturing the specific organism. A direct polymerase chain reaction assay of a clinical sample may provide a more rapid test result than a culture, but the assay is less sensitive (31–43 % sensitivity comparing to culture), especially when performed in blood [11]. A serological test alone is considered inadequate to confirm the diagnosis, especially in endemic regions where the background seropositivity rate can be more than 50 %. For example, in certain endemic regions such as Thailand, the diagnosis of active melioidosis needs a high antibody titre (i.e. >1:640) or a fourfold rise in the indirect haem-agglutination assay (IHA) titres in convalescent samples [2, 7, 12].

We report a healthy young female who presented with acute pulmonary melioidosis with septicaemia. She had bilateral multiple pulmonary cavitary lesions in all three zones of the lung. The diagnosis was confirmed by broncho-alveolar lavage fluid culture positivity for melioidosis with very high antibody titres.

## Case presentation

This 29 years old female presented with 1 week history of low grade intermittent fever with constitutional symptoms and a persistent worsening of cough for the same duration. Her fever did not associate with chills or rigors and she did not have hemoptysis. She was previously healthy and she was a housewife who engaged herself actively in gardening at her own backyard. She did not have a contact history or a past history of tuberculosis. She had no history of foreign travel. She did not have any other symptoms to note. On examination, she was average built with a BMI of 24 kg/m^2^. She was febrile. She was neither pale, nor she had lymphadenopathy or organomegaly. Her pulse was 120 bpm and blood pressure was 86/60 mmHg on admission. There were few scattered crackles in her lungs. There were no signs of focal consolidation, effusions evident on examination. She did not have skin ulcers, cellulitis or skin abscesses. She had no features of meningism, focal neurological deficits and had normal fundi.

**Fig. 1:**
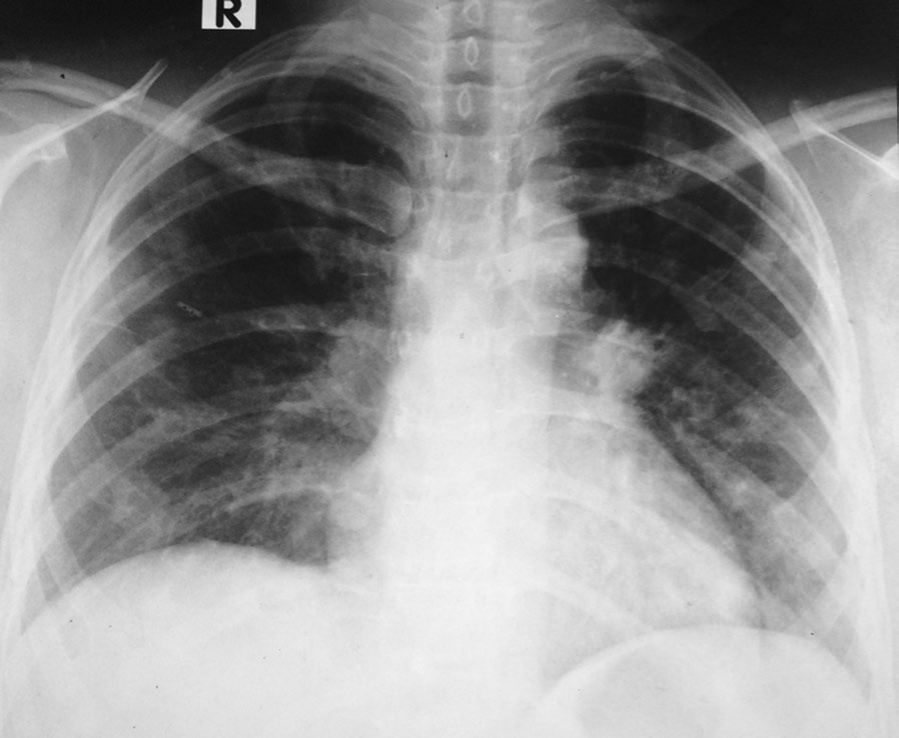
Chest radiograph with bilateral patchy shadows involving both lung fields

**Fig. 2:**
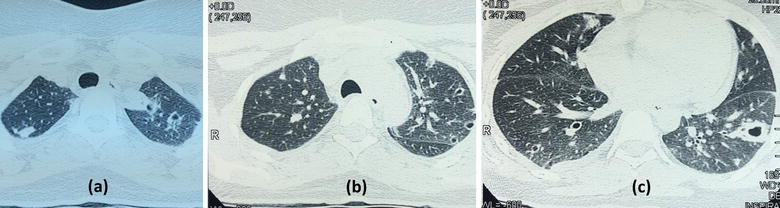
HRCT of Upper (**a**), Middle (**b**), Lower (**c**) Zones of the lungs showing multiple bilateral cavitary lesions without air-fluid levels

Her investigations showed a haemoglobin of 11.2 g/dl with neutrophil leukocytosis (total 18,000/µl with 82 % neutrophils) with platelet count of 540,000/µl. Her ESR was 60/1st h and CRP was 458 µ/l (<6). Chest radiograph showed bilateral patchy shadows involving both lung fields (Fig. 1). Her high-resolution computed tomography (HRCT) scan showed multiple bilateral cavitary lesions in the upper, middle and lower zones of lungs without air-fluid levels (Fig. 2). We administered broad spectrum intravenous antibiotics (Ceftriaxone 2 g 12 h and Clindamycin 600 mg 8 h) for 5 days, in spite of which she continued to have high fever spikes and showed clinical deterioration. Her blood and urine cultures revealed negative results. Since her Mantoux test and sputum smear and culture for acid fast bacilli (AFB) were negative, we proceeded to perform an early fibro-optic bronchoscopy. Bronchoscopy was normal and the broncho-alveolar lavage fluid (BALF) specimens were negative for AFB, *Pneumocystis jiroveci* and fungi. All BALF cultures including bacterial, fungal and mycobacterial were performed and special cultures were requested for melioidosis due to poor response to initial broad spectrum antibiotics. Surprisingly, culture for melioidosis using Ashdown’s agar became positive for *Burkholderia pseudomallei*, while all other cultures were negative. On Ashdown’s agar, the colonies showed typical purple, wrinkled morphology after 72 h of incubation. Melioidosis antibody titre (Indirect Haem-agglutination Assay) was detected to be highly positive at a titre of 10,640. Her transthoracic and trans-esophageal echocardiogram did not show vegetations. Autoimmune panel with ANA and ANCAs were negative. Contrast CT chest and abdomen did not reveal any visceral lymphadenopathy or abscesses. Her HIV, TPHA/VDRL, HbA1c (5.5 %), renal, liver functions and serum immunoglobulin levels were all normal.

After detecting BALF cultures and antibody titre positivity for Melioidosis, we changed the antibiotics to high dose intravenous Meropenem (2 g 8 h). She showed excellent response for these antibiotics within few days and demonstrated a marked clinical improvement and radiographic resolution. After 7 days of intravenous antibiotics, melioidosis antibody titers reduced to 2750 and subsequently to 160 after 4 weeks.

We continued her intravenous antibiotics for a month and discharged her on oral Doxycycline (100 mg 12 h) for another 24 weeks. In review of the patient, her recovery was uncomplicated after completion of the course of antibiotics.

## Discussion

Melioidosis is a noteworthy potentially fatal infectious disease which has been recently observed in increasing incidence, at an alarming rate. This observation may be due to the true increase in number of cases and/or due to improved awareness and diagnostics. Availability of specific culture methods and serology has enabled early confirmation of this disease. Melioidosis, if not promptly treated early with appropriate antimicrobials, it could lead to fatality. Therefore, high degree of suspicion is necessary to request specific investigations to guide the specific management strategies.

Our patient presented with an acute pulmonary involvement with cavitary lesions in all three zones of the lung with an impending septic shock state. After taking the initial cultures, she was started on intravenous broad spectrum antibiotics for which she showed a poor response. Due to her further deterioration, urgent fibre-optic bronchoscopy was performed and BALF cultures for conventional organisms, mycobacteria, fungi and organism causing melioidosis were sent. Although standard culture media did not show a growth of organisms, a special culture media (Ashdown’s agar) which is usually used for respiratory tract specimens showed growth of the organism causing melioidosis.

Intravenous Meropenem and Ceftazidime are recommended to be used for at least 14 days, until the acute episode is settled. There after Co-trimoxazole, Co-amoxiclav or Doxycycline could be used for subsequent 6–12 months to prevent recurrence [13]. We used intravenous high dose Meropenem (2 g 8 h) initially and continued her on oral Doxycycline (100 mg 12 h) for 6 months.

In her case, she had no co-morbidities and did not have any identifiable risk factors to contract melioidosis. It is exceedingly uncommon for melioidosis to occur in young healthy adults, especially the acute fulminant form. Even though melioidosis is mostly described to occur in forest areas and paddy cultivation areas in endemic countries [1], our patient may have acquired the disease through home gardening.

The presentation of bilateral cavitary lesions in all lobes of lung is described as an uncommon finding in melioidosis, as most of the time it has a predeliction to the upper lobes of the lung [9, 10]. But, a study done in Thailand in late 1980s has described that disseminated nodular lesions are seen in 84 % of patients with acute septicaemia phase [14]. Since melioidosis is a culture based diagnosis, obtaining microbiological samples in all relevant samples is mandatory and using special culture media when initial cultures become negative is of paramount importance. Chan et al. described a patient presenting with right lower zone lung cavity with mediastinal lymphadenopathy who isolated the organism by Endo-bronchial ultrasound and fine needle aspiration of the mediastinal lymph nodes. In this patient, both blood and BALF cultures for melioidosis were negative [15]. Isolation of the organism in our case was achieved by performing an early bronchoscopy and sending BALF cultures. This demonstrates the importance of looking for more relevant microbiological samples and using special culture media in diagnosis of melioidosis, even when the initial cultures were negative.

## Conclusions

It is of paramount importance for physicians to clinically suspect melioidosis even in patients without underlying risk factors, especially when poor response is observed for conventional antibiotics. Bilateral multiple cavitary lung lesions involving all three zones of the lung is an uncommon presentation of pulmonary melioidosis. Even if the initial conventional cultures become negative in pulmonary melioidosis, planning early bronchoscopy and broncho-alvelolar lavage would be useful to obtain microbiological samples. An exceedingly high titre of serum antibodies to melioidosis is very helpful in supporting the diagnosis.
